# Supplementary material for: Building a Chinese pan-genome of 486 individuals
Source: Commun Biol. 2021 Aug 30;4:1016. doi: 10.1038/s42003-021-02556-6 (PMC8405635; doi:10.1038/s42003-021-02556-6)
Supplement: Supplementary file 2 — Supplementary Information [file 42003_2021_2556_MOESM2_ESM.pdf]

Building a Han Chinese pan-genome of 486 individuals

Qiuhui Li, Shilin Tian, Bin Yan, Chi Man Liu, Tak-Wah Lam, Ruiqiang Li, Ruibang Luo

Supplementary Materials

SUPPLEMENTARY FIGURES 2

SUPPLEMENTARY FIG. 1. REFSEQ PROTEIN FAMILY ANNOTATION OF ALL NOVEL SEQUENCES. 2
SUPPLEMENTARY FIG. 2. PFAM PROTEIN FAMILY ANNOTATION OF THE PLACED SEQUENCES. 2
SUPPLEMENTARY FIG. 3. REPETITIVE SEQUENCE ANNOTATION. 3
SUPPLEMENTARY FIG. 4. GC CONTENT (%) OF THE NOVEL SEQUENCES. 4
SUPPLEMENTARY FIG. 5. THE SIZE OF NOVEL SEQUENCES OF THE 90 INDIVIDUALS FROM BGI (AFTER REMOVING CONTAMINATIONS). 4
SUPPLEMENTARY FIG. 6. PATHOGENICITY SCORE DISTRIBUTION OF THE INSERTION POINTS IN REGULATORY REGIONS. 5
SUPPLEMENTARY FIG. 7. VALIDATION OF THE PREDICTED CODING GENES. 6
SUPPLEMENTARY FIG. 8. THE SIZE OF NOVEL SEQUENCES OF THE 90 INDIVIDUALS FROM BGI (BEFORE REMOVING CONTAMINATIONS). 6
SUPPLEMENTARY FIG. 9. PERCENTAGE OF THE NOVEL SEQUENCES OF THE 90 INDIVIDUALS THAT WERE ALIGNED TO THE CPG COMMON SEQUENCES. 7
SUPPLEMENTARY TABLE 1. NOVEL SEQUENCE ENRICHMENT STATISTICS IN THE SIX MAIN REPETITIVE SEQUENCE TYPES. 8
SUPPLEMENTARY TABLE 2. NOVEL SEQUENCE ENRICHMENT STATISTICS IN THE SIX MAIN REGULATORY REGIONS. 8
SUPPLEMENTARY TABLE 3. CENTROMERIC SEQUENCES IN CPG. 9
SUPPLEMENTARY TABLE 4. THE PATHOGENICITY SCORES OF THE INSERTION POINTS IN CTCF BINDING SITES AND PROMOTERS. 9
SUPPLEMENTARY TABLE 5. EFFECTS OF DIFFERENT COMMON SEQUENCES ON VARIANT CALLING. 10

SUPPLEMENTARY NOTES 11

NOTE 1: DETECTION OF CONTAMINANTS 11
NOTE 2: EVALUATING AMOUNT OF COMMON SEQUENCES FOR LARGE POPULATIONS 11
NOTE 3: MUTATION RATE NEAR INSERTION POINTS 12
NOTE 4: ENRICHMENT OF THE NOVEL SEQUENCES IN GRCH38 12
NOTE 5: ANALYSIS OF THE NOVEL SEQUENCES 13
NOTE 6: PREDICTION AND VALIDATION OF NOVEL GENES 13
NOTE 7: APPLICATIONS OF THE COMMON SEQUENCES 14
NOTE 8: ASSEMBLY OF THE NOVEL SEQUENCES OF 90 HAN CHINESE 14
NOTE 9: VALIDATION OF THE COMMON SEQUENCES IN 90 HAN CHINESE 15
NOTE 10: COMMANDS AND PARAMETERS 15

SUPPLEMENTARY METHODS 18

MERGING OF SINGLE-END-PLACED CONTIGS. 18
ADMIXTURE ANALYSIS. 18

SUPPLEMENTARY REFERENCES 19

Supplementary Figures

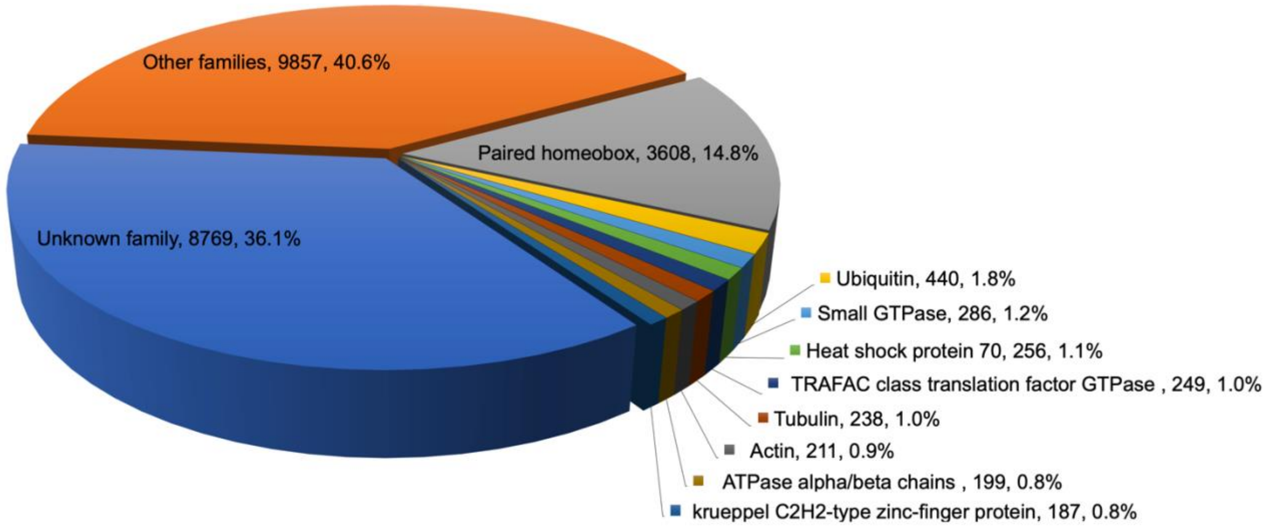

Supplementary Fig. 1. RefSeq protein family annotation of all novel sequences. The Refseq proteins were downloaded and aligned to all novel sequences by tBlastN. Only the best hit was recorded for each sequence.

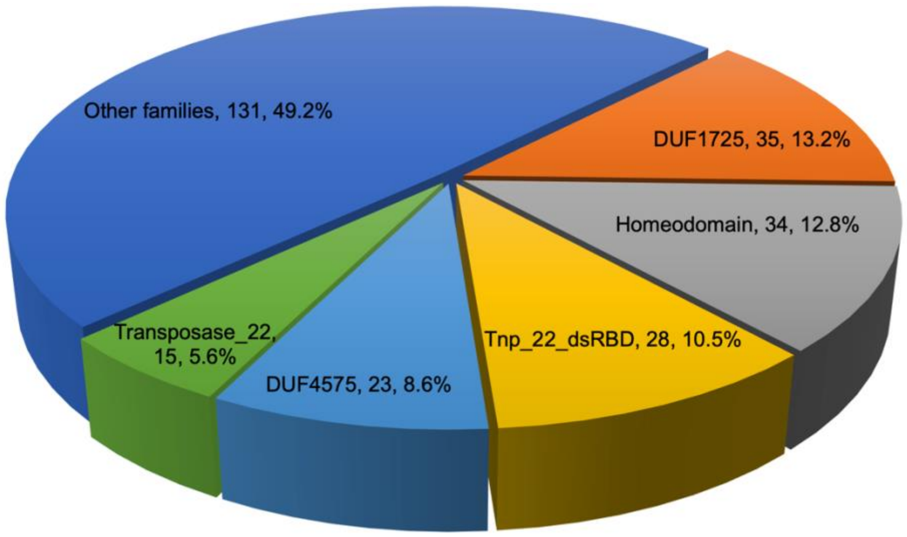

Supplementary Fig. 2. Pfam protein family annotation of the placed sequences. The Pfam database was downloaded and aligned to the placed sequences using PfamScan. Only the best hit was recorded for each placed sequence.

A

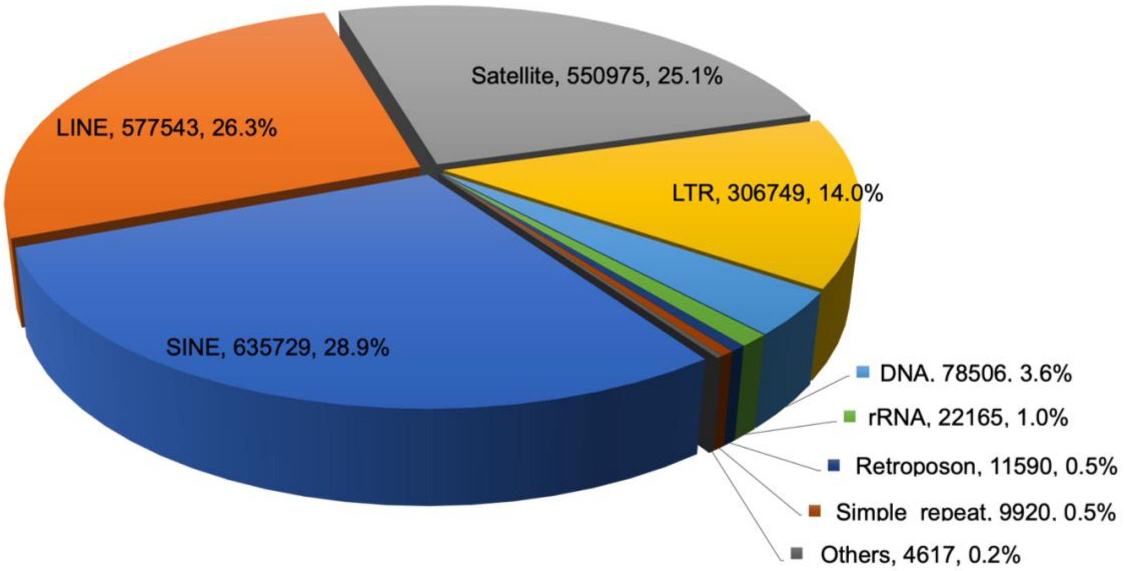

B

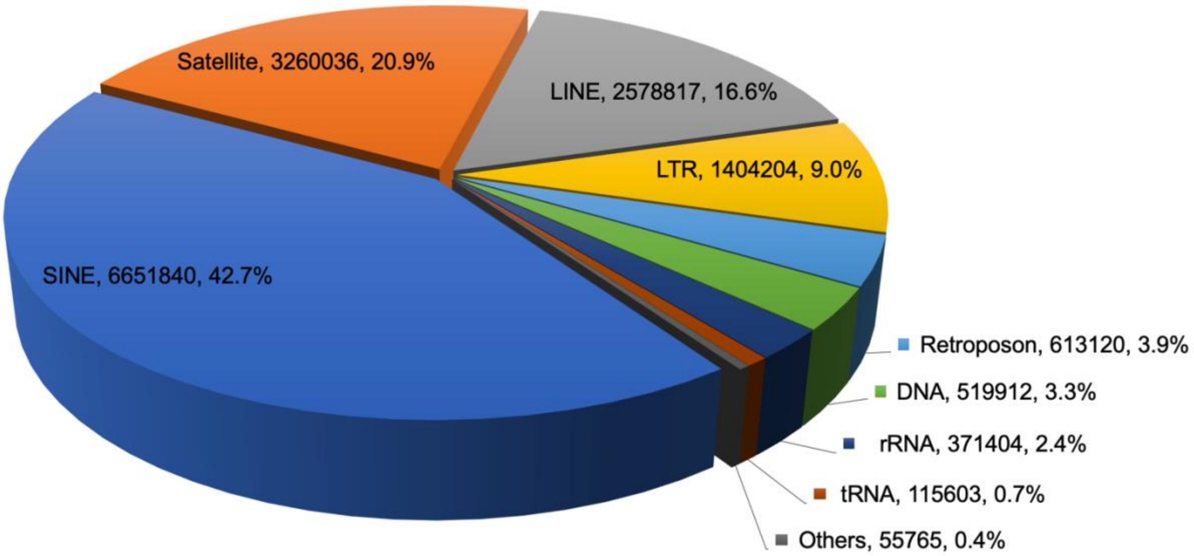

Supplementary Fig. 3. Repetitive sequence annotation. The figure shows the number of the placed sequences (A), and the unplaced sequences (B) being identified as different types of repetitive sequences. 30% of the placed sequences and 5.8% of the unplaced sequences were identified as repetitive sequences. SINE, LINE, and satellite are three main types of repetitive sequences.

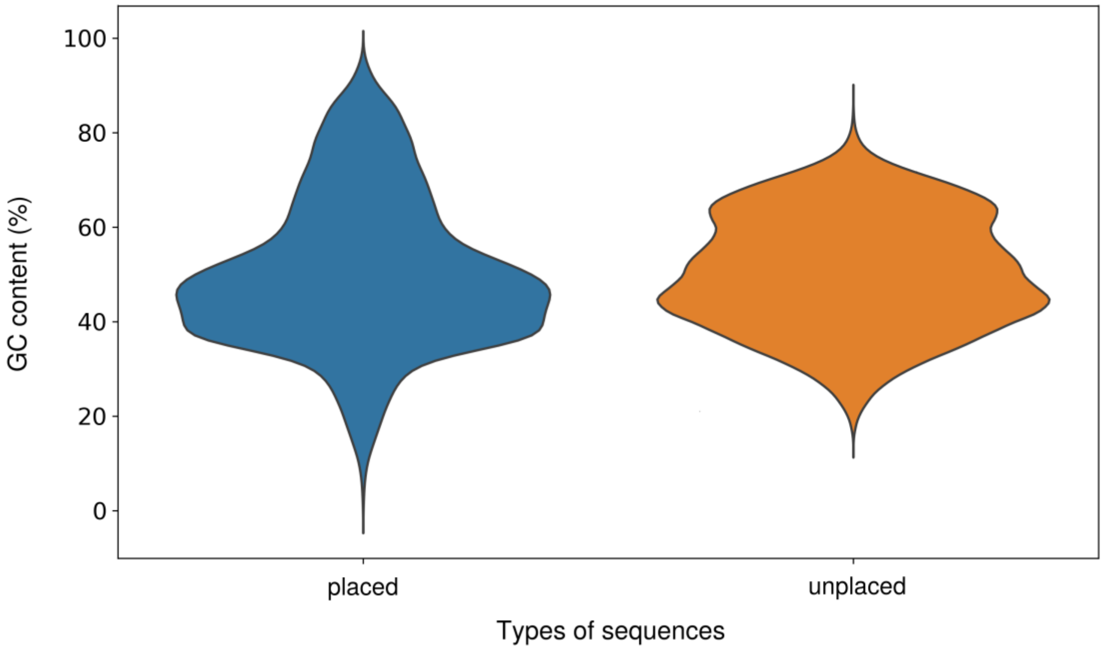

Supplementary Fig. 4. GC content (%) of the novel sequences. A violin plot showing the distribution of GC content of the novel sequences.

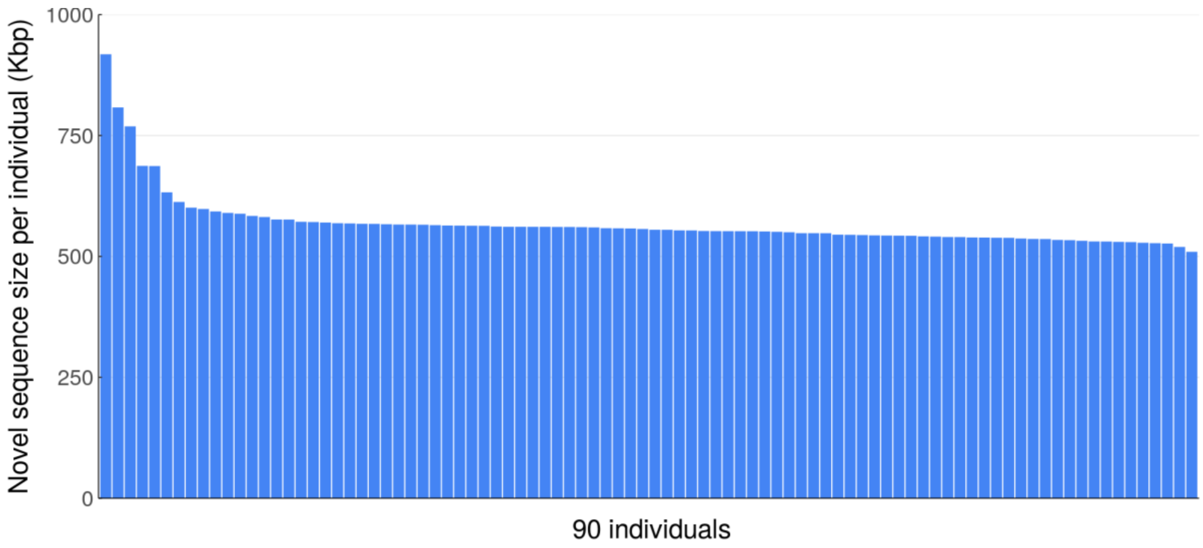

Supplementary Fig. 5. The size of novel sequences of the 90 individuals from BGI (after removing contaminations). Each column in X-axis represents an individual. Y-axis indicates the size of novel sequences identified from the 90 Han Chinese.

A

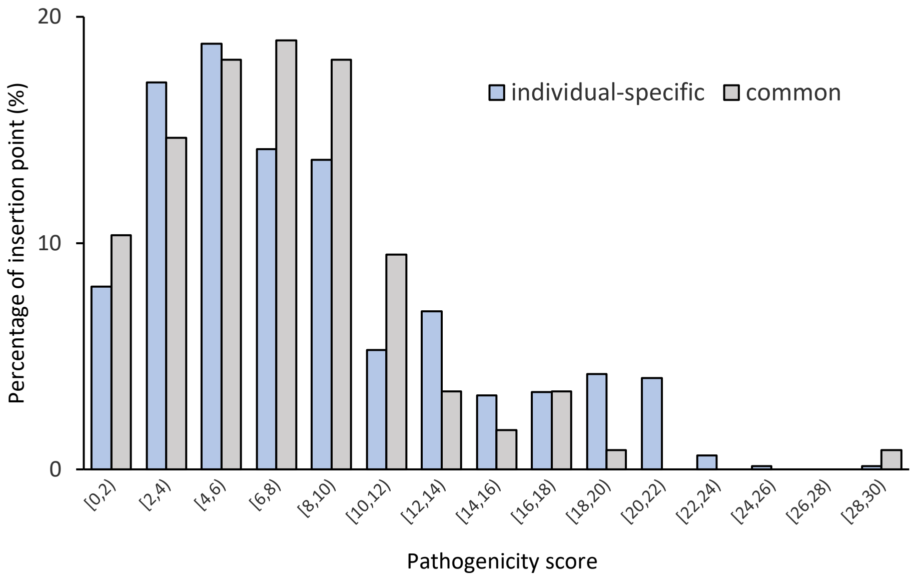

B

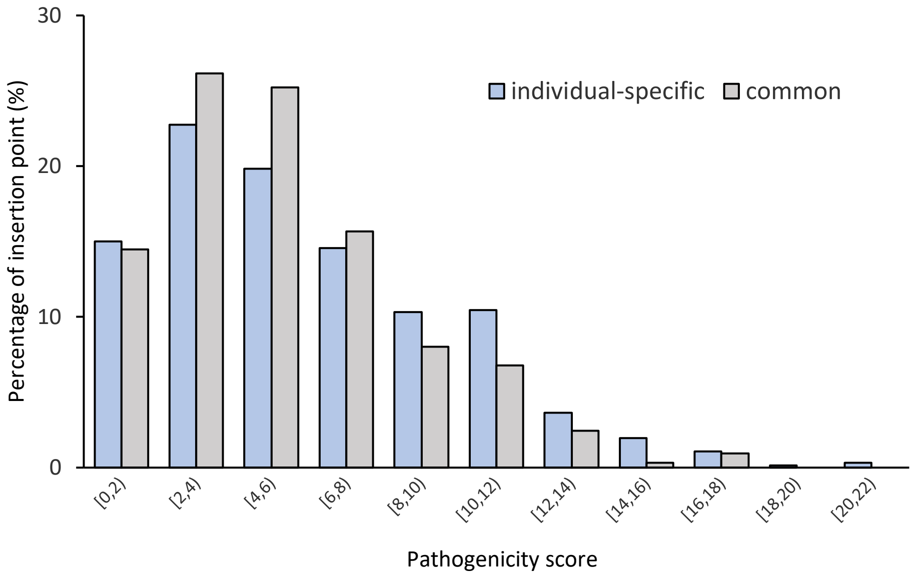

Supplementary Fig. 6. Pathogenicity score distribution of the insertion points in regulatory regions. Y-axis is the percentage of insertion points of the placed sequences. X-axis is the binning of pathogenicity scores. A, Insertion points in gene promoters. B, Insertion points in CTCF binding sites.

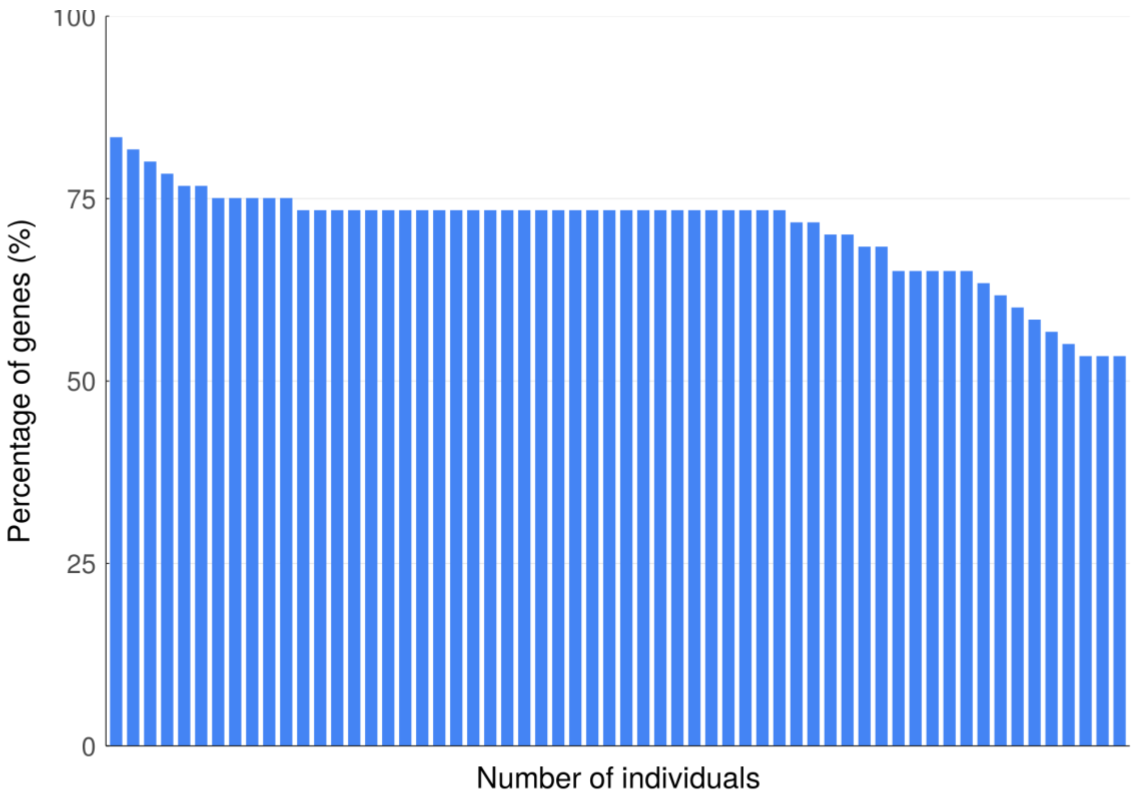

Supplementary Fig. 7. Validation of the predicted coding genes. There were 53 coding genes predicted from the novel sequences. For validation, we aligned the RNA-seq data of 60 HapMap CEU individuals to the 53 coding genes. The Y-axis shows the percentage of the 53 predicted coding genes that were found in at least corresponding number of individuals shown in X-axis.

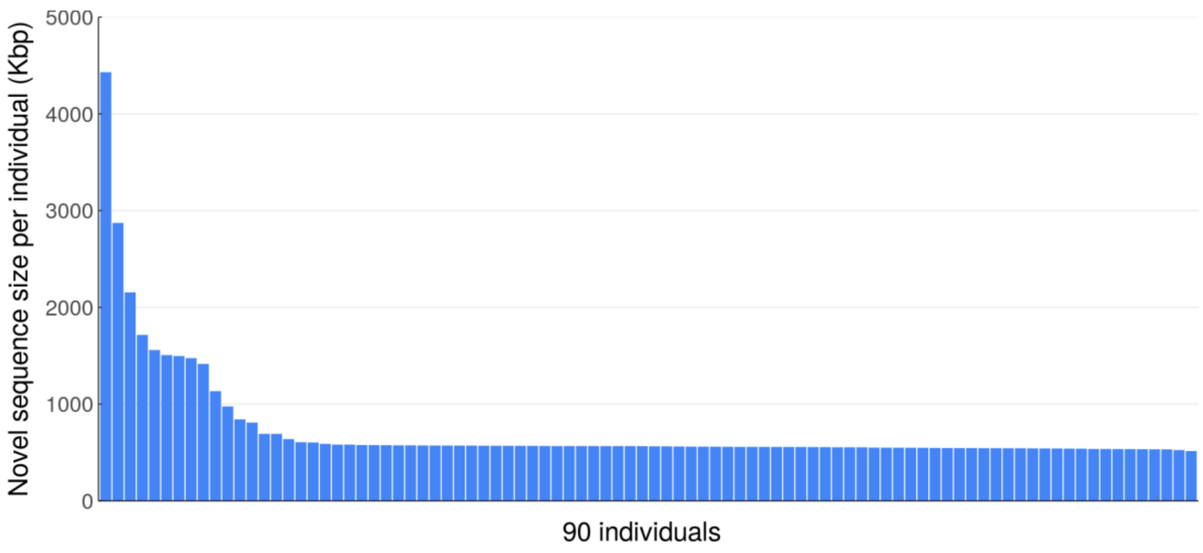

Supplementary Fig. 8. The size of novel sequences of the 90 individuals from BGI (before removing contaminations). Each column in X-axis indicates every individual of the 90 Han

Chinese. To highlight a smaller size of novel sequences in these 90 individuals (Y-axis), we used the identified sequences before removing contaminations. While most of the individuals are below 1Mbp, there are a few above because of unremoved contaminations.

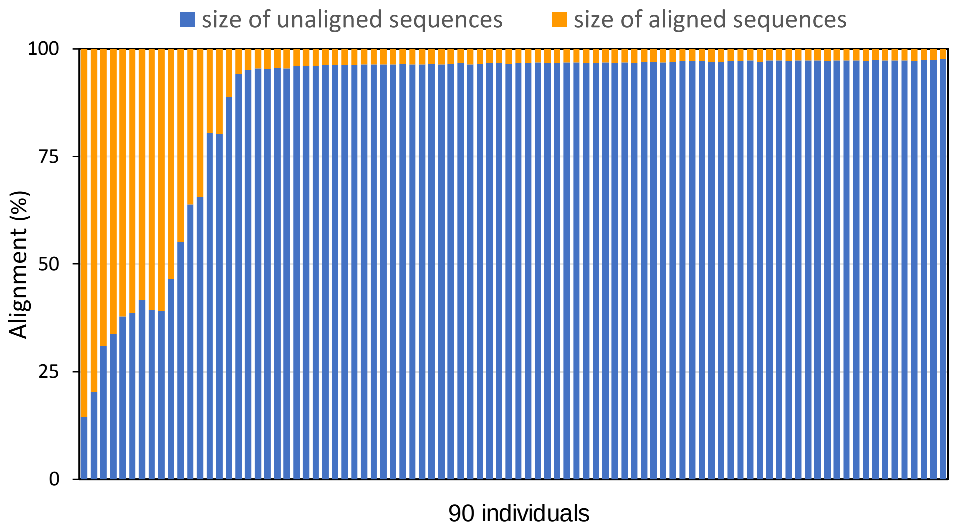

Supplementary Fig. 9. Percentage of the novel sequences of the 90 individuals that were aligned to the CPG common sequences. Y-axis indicates the percentage of the novel sequences (before removing contaminations) of the 90 Han Chinese individuals from BGI aligned to the CPG common sequences. X-axis indicates every individual of the 90 Han Chinese.

Supplementary Tables

Supplementary Table 1. Novel sequence enrichment statistics in the six main repetitive sequence types.

Type	Number of insertion points	Percentage in GRCh38	Z-Score
LINE	930 (6.61%) [a]	20.67%	-9.88
SINE	1867 (13.27%)	12.49%	0.70
LTR	449 (3.19%)	8.76%	-3.89
DNA	116 (0.82%)	3.53%	-1.53
Satellite	1421 (10.10%)	2.37%	4.52
Simple repeat	3980 (28.29%)	1.35%	8.16

The table shows the number and percentage of insertion points in six main repetitive sequence types. “Low complexity repeats” were included in “Simple repeat”. We computed a Z-Score for each type by testing the observed number of the insertion points against the expected number if the insertions are random to assess for enrichment or depletion of insertion points in different types. While the probability of randomly selecting a score between -1.96 and +1.96 standard deviations from the mean is 95%, we consider a Z-score above 1.96 as a significantly enriched and below -1.96 as significantly depleted.

Supplementary Table 2. Novel sequence enrichment statistics in the six main regulatory regions.

A

Type	Number of insertion points	Percentage on GRCh38	Z-Score
CTCF binding site	1,021 (7.3%)	2.92%	3.23
Promoter	772 (5.64%)	2.42%	2.35
Enhancer	210 (1.53%)	2.63%	-0.86
Promoter flanking region	1,033 (7.55%)	8.61%	-0.92
TF binding site	96 (0.70%)	0.38%	0.25
Open chromatin region	179 (1.31%)	1.19%	0.10

B

Type	Number of insertion points	Z-Score
CTCF binding site	355 (7.85%)	3.06
Promoter flanking region	349 (7.72%)	-0.53
Promoter	128 (2.83%)	0.25
Enhancer	83 (1.84%)	-0.42
Open chromatin region	52 (1.15%)	-0.02
TF binding site	39 (0.86%)	0.30

C

Type	Number of insertion points	Z-Score
CTCF binding site	666 (7.27%)	3.01
Promoter flanking region	684 (7.47%)	-0.87
Promoter	644 (7.03%)	3.03
Enhancer	129 (1.41%)	-0.79
Open chromatin region	127 (1.39%)	0.15
TF binding site	57 (0.62%)	0.18

The table shows the number and percentage of insertion points in six main regulatory regions. We computed a Z-Score for each type by testing the observed number of the insertion points against the expected number if the insertions are random to assess for enrichment or depletion of insertion points in different regions. A, B and C show the results of "all novel sequences", "common sequences" and "individual-specific sequences", respectively. While the probability of randomly selecting a score between -1.96 and +1.96 standard deviations from the mean is 95%, we consider a Z-score above 1.96 as a significantly enriched and below -1.96 as significantly depleted.

Supplementary Table 3. Centromeric sequences in CPG.

Sequence type	Total size of centromeric sequences	Size of alphoid sequences	Size of hsat2,3 sequences
Common	211,713 (0.5%)	92,668 (0.2%)	119,045 (0.3%)
Individual-specific	1,624,428 (0.7%)	603,240 (0.3%)	1,021,188 (0.4%)

The table shows the size and percentage of centromeric satellite repeat sequences that were identified by using dna-brnn method based on the common and individual-specific sequences of CPG. The centromeric sequences consist of two types, hsat2,3 and alphoid.

Supplementary Table 4. The pathogenicity scores of the insertion points in CTCF binding sites and Promoters.

Regulatory type	Insertion points of common sequences	Insertion points of individual-specific sequences
CTCF binding site	5.20	5.91
Promoter	7.31	8.18

The table shows the average pathogenicity scores of the insert points of the common and individual-specific sequences of CPG in CTCF binding sites and promoters.

Supplementary Table 5. Effects of different common sequences on variant calling.

Reference Genome	$N_{True\text{-}pos}$	$N_{False\text{-}pos}$	$N_{False\text{-}neg}$
GRCh38	3,249,098	9,895	59,116
GRCh38 + Common (≥2 individuals [a])	3,249,085	9,427	59,129
GRCh38 + Common (≥5 individuals)	3,249,088	9,573	59,126
GRCh38 + Common (≥10 individuals)	3,249,089	9,625	59,125
GRCh38 + Common (≥20 individuals)	3,249,091	9,618	59,123
GRCh38 + Common (≥2 individuals & repetitiveness<50% [b])	3,249,084	9,444	59,130
GRCh38 + Common + Individual-specific sequences	3,249,089	9,291	59,125

The table shows the effect of different common sequences generated by [a] minimal number of individuals who shared the sequences in CPG on variant calling. [b] the repeat sequences were identified by RepeatMasker. $N_{True\text{-}pos}$= the number of true-positive variants. $N_{False\text{-}pos}$=the number of false-positive variants. $N_{False\text{-}neg}$= the number of false-negative variants.

Supplementary Notes

Note 1: Detection of contaminants

We identified contaminated sequences by aligning the novel sequences from the 486 Chinese individuals to the non-cordate sequences (provided by Centrifuge[1]) by BLASTN. To detect whether there were significant contaminants in our individual samples, we first classified the mapped novel sequences at the species level. For each type of contaminated sequence, we counted the total number in 486 individuals, denoted N_c and obtained the largest number of sequences that a person carried, denoted S_c. We divided S_c by N_c to determine whether some contaminated sequences were concentrated on a sample. There were no contaminants with both high number and high percentage (Supplementary Data 4), indicating no significant contaminants among the 486 individuals. Additionally, it was noteworthy that some sequences mapping to Brevundimonas should not be in human genomes. We reasoned that it might be because the genome of Brevundimonas was contaminated by human DNA, supported by the previous study, human DNA contaminating genomes of other species [2].

After building the Chinese pan-genome, we aligned CPG sequences to the Nucleotide dataset by BLASTN and found that only 0.3% of CPG (0.754 Mbp) had good alignments (≥95% coverage of each other) with the nonhuman sequences. The result indicates that our novel sequences were not contaminated.

Note 2: Evaluating amount of common sequences for large populations

To understand why the total size of common sequences is determined by the occurrence frequency (OF), we consider a large population of N individuals. Let s be a sequence and f_s be the number of individuals in the population carrying s. Suppose that we use an OF of p ($0 \le p \le 1$). Now, we uniformly sample n individuals from the population. The expected number of individuals in this sample carrying sequence s is nf_s/N. Hence, according to the definition of OF, s will be a common sequence in this sample if and only if $nf_s/N > np$, in other words $f_s/N > p$. Note that this condition does not depend on the sample size n. Therefore, the set of common sequences defined by a particular OF is independent of the sample size, given that the sampling from original population is unbiased.

On the other hand, we claim that the total size of common sequences is unbounded under constant occurrence threshold (absolute number rather than percentage) as the population size increases. To explain the intuition behind our claim, we use a simplified tree model for population growth. We model the generations of a population as levels of a tree. The population starts from a single person, represented by the root node of the tree, in level 0. The root's offspring is represented by its child nodes in level 1. The offspring of a level 1 node is represented by its child nodes in level 2 and so on. For ease of explanation, we assume the tree to be a full binary tree, i.e. each non-leaf node has exactly two children. Suppose that there are m levels (0, 1, ..., m-1). The number of nodes in level k is then 2^k and the total number of nodes in the tree is N=2^{m-1}.

We also use a simplified model for sequence inheritance. We assume that each node carries a fixed number of sequences, which are then inherited by its children with a certain probability. In addition to sequences inherited from its parent, each node also has its novel

sequences not found in its ancestors. We say that a sequence "originates" from a node if it is that node's novel sequence. We further assume that all novel sequences are unique, i.e. no two nodes will share the same novel sequence by chance. Under this model, a sequence originating from some node *x* can only be inherited by nodes within the subtree rooted as *x*. As an example, a sequence with 100% occurrence in the population must originate from the root (level 0); a sequence with 50% occurrence originates from either of the root's children (level 1); a sequence with 25% occurrence originates from one of the level 2 nodes and so on. On the other hand, a sequence originating from a leaf node (level m-1) has occurrence 1 (not 1%); a sequence originating from a leaf's parent node (level m-2) has occurrence 2 and so on.

Now let us examine common sequences defined using occurrence threshold (absolute number). For example, using an occurrence threshold of 4, the common sequences will be those originating from levels 0 to m-3. The total number of nodes in these levels is 2^{m-2}-1, which is roughly *N*/4. Now consider doubling the population by introducing a new level m with 2^m people. With an occurrence threshold of 4, the common sequences will be those originating from levels 0 to m-2. The total number of nodes in these levels is 2^{m-1}-1, which is roughly *N*/2. Hence the number of common sequences also doubles given the same occurrence threshold.

Next, we examine common sequences defined using occurrence frequency (OF). For example, using an occurrence frequency of 12.5%, the common sequences will be those originating from levels 0 to 3, which contain only 15 nodes. After doubling the population, the common sequences for 12.5% OF will still be those from levels 0 to 3. Hence the number of common sequences remains unchanged given the same OF.

In summary, if we use a constant occurrence threshold, the common sequence size has a linear relationship with the population size, therefore it is unbounded as the population grows. However, if we use a constant OF, the common sequence size is invariant of the population size, which matches our observation in the "Result" section.

Note 3: Mutation rate near insertion points

We used dbSNP version 151 to determine the mutation rate of the genome positions adjacent to the insertion points [3]. The mutation rate of the whole genome was calculated as the number of genome positions with at least an SNP reported over the reference genome size. For each insertion point in the common or individual-specific sequences, we determined the mutation rate of 500 flanking base pairs.

Note 4: Enrichment of the novel sequences in GRCh38

To explore whether the novel sequences were enriched with the repeat regions, we downloaded the repeat dataset of RepeatMasker. We calculated the average percentage P_{ref_rpt} of each type of repeat sequences on GRCh38, P_{ref_rpt} = the length of repeat sequences/length of the primary assembly GRCh38 sequences. Then, we calculated the percentage P_{ins_rpt} of insertion points falling within a type of repeat region, P_{ins_rpt}= The number of insertion points located in the corresponding repeat regions / the total number of insertion points. The P_{ref_rpt} and P_{ins_rpt} were used to calculate the Z-score. If the Z-score exceeds 1.96, it suggests that the novel sequences are significantly enriched with that type of repeat region (Supplementary Table 1).

We next analyzed the frequency of the novel sequences inserted to the regulatory regions using the same method as above. We extracted the regulatory dataset from Ensembl (Version: 20190329, GRCh38). Because no regulatory annotations of ChrY are reported in the dataset, we only considered functional elements on autosomes and ChrX. The result shows enrichment of the novel sequences with CTCF binding sites and promoter regions (Supplementary Table 2).

Given that mutations in regulatory regions could affect gene expression, we evaluated pathogenicity scores of the insertion points in CTCF binding sites and promoters. The pathogenicity score was an average of six C scores of the insertion point and the points before the insertion site. The insertion points of the individual-specific sequences held higher pathogenicity than that of the common ones (Supplementary Fig. 6). Noticeably, the score 8.18 on promoters was obviously higher than the average 4.83 of GRCh38 (Supplementary Table 4).

Note 5: Analysis of the novel sequences

We used VEP (release 98) to annotate the placed sequences and to detect structural variants in the 1000 Genomes Project (Phase 3) that overlapped with our insertion regions (Supplementary Data 6). We aligned all sequences to RefSeq human proteins with BlastX and mapped the placed sequences to the Pfam 32.0 dataset by PfamScan version 1.6 [4] (Supplementary Data 7 and 8). In addition, we identified the repeat sequences by RepeatMasker and calculated the proportions of different repeat types of sequences relative to the total placed and unplaced sequences (Supplementary Data 9).

We examined whether the novel sequences of three pan-genomes CPG, APG and HCPG were detected previously. Their genomic sequences were mapped to the decoy sequences hs38d1 (5.79 Mbp) using BWA. There were 16.6 Mbp, 4.6 Mbp and 4.1 Mbp of APG, CPG and HCPG, respectively, mapped to hs38d1 with at least 90% identity and 80% coverage. Comparatively, the alignment percentage 1.67% of CPG was the lowest. APG likely contained redundant sequences and therefore resulted in a large 16.6 Mbp length mapped to only 5.79 Mbp of hs38d1.

Note 6: Prediction and validation of novel genes

We downloaded the EST human dataset and protein sequences from NCBI and predicted protein-coding genes by MAKER (version 2.31.10) [5]. The repetitive regions of the novel sequences were identified and masked in advance. Based on EST and protein datasets, MAKER directly produced gene annotations. Then, we trained the *ab initio* gene predictor. The predicted genes with incomplete CDS regions were discarded. We finally obtained 53 protein-coding genes annotated with the novel sequences.

In addition, we mapped RNA sequences of 60 CEU individuals [6] to transcripts of the 53 genes with HISAT2 (version 2.1.0) [7]. If a transcript had an alignment with at least 90% identity and 95% coverage, the gene was considered as validated. Among 53 predicted genes, 50 (94.3%) were verified in at least one CEU individual, whereas 32 were validated in all individuals. The result indicates high confidence of our prediction (Supplementary Fig. 7).

Note 7: Applications of the common sequences

To examine the effect of the common sequences on mapping, we aligned the reads that could not be aligned to GRCh38 but could be aligned to their own novel sequences, to the common sequences by BWA-mem. When calling variants (SNPs and indels), we first prepared a new reference by combining the common sequences with the full GRCh38, including all unplaced sequences, alternative sequences, decoy sequences and HLA subtype sequences. Illumina sequencing reads (60x and 300x HG005, 60x HG001 and 60X HG002) from the Genome In A Bottle (GIAB) project [8] were aligned to the new reference by BWA. Next, we used Picard to remove reduplicates [9] and called variants by GATK HaplotypeCaller (Version: 4.1.3.0). The identified variants in the primary GRCh38 sequences were compared to the truth dataset. The results of variant calling were evaluated. In addition, to investigate which part of the novel sequences (the less or highly repeated ones) played a vital role in decreasing the false-positive variants, we identified repetitive parts of the common sequences by RepeatMasker and removed sequences with ≥ 50% repeat percentages. After the remaining sequences were added to GRCh38 as a new reference, we did re-call variants on 60x HG005. Since the accuracy of variant calling remained basically unchanged, we considered that the less repeated sequences could be the key to realize a better performance of variant calling.

To assess whether the common sequences shared by more than one individual were the most suitable for variant calling, we added the whole CPG (7 times larger than the common sequence size) to GRCh38. Only 136 false-positive variants were eliminated. Consequently, we constructed the new reference sequences by combining the common sequences shared by ≥5, ≥10 and ≥20 individuals with GRCh38, respectively and then aligning 60x HG005 reads to these reference sequences for calling variants. Comparatively, the sequences shared by ≥ 2 individuals can decrease the largest number of false-positive variants (Supplementary Table 5).

Note 8: Assembly of the novel sequences of 90 Han Chinese

To guarantee the completeness of the novel sequences to the largest extent, we did not use the provided scaffolds of 90 people assembled by SOAPdenovo2. We first aligned the raw reads of 90 Han Chinese to GRCh38 and then assembled the unaligned reads into the sequences by using MEGAHIT due to its integrity and continuity of assembling. All assembled sequences were mapped to GRCh38.p13 by BWA-mem. Sequences aligned with ≥ 90% identity and ≥ 80% coverage were filtered out. Finally, we removed contaminants identified by Kraken2 [10] (confidence score threshold 0.05) and sequences mapped to EmVec and UniVec sequences with ≥ 80% identity and ≥ 50% coverage.

We found that the average size of the novel sequences per person in the 90 Han genomes was smaller than novel DNA size in the 486 individuals (Supplementary Fig. 8). It may be related to the polymerase chain reaction (PCR) amplification. PCR amplification performs well when the GC content ranges from 37% to 42%. But if sequences are from genomes with a high or low GC content, the genome assembly is more likely to be incomplete [11]. However, our result shows that the GC content of the novel sequences can reach up to 49%, much higher than the optimal range of GC content in PCR amplification. The excessive GC content could cause partial missing of sequencing reads when performing PCR amplification, which would lead to the incomplete assembly of novel sequences. Therefore, we suggest using PCR-free Illumina sequencing data when assembling novel sequences.

Note 9: Validation of the common sequences in 90 Han Chinese

We aligned the novel sequences of 90 Han samples to our common sequences by BLASTN and found that 77 individuals had more than 80% of novel sequences aligned with 80% identity (Supplementary Fig. 9). Because the remaining 13 individuals had abnormally large novel sequence sizes and low alignment rates, we next tried to examine the reason why these novel sequences were not mapped to our common sequences (named unknown sequences). First, we chose a representative sample (HG00427) which had the lowest alignment percentage and the largest novel sequence size and aligned 3,986 unknown sequences of this sample to the NCBI Nucleotide (NT) database. We found only 173 sequences mapped with ≥80% identity, suggesting that most of the unknown sequences do not belong to existing genomes. Then, we aligned the unknown sequences of one of the 13 individuals to those of the remaining 12 individuals using BLASTN. There were about 91.4% of the unknown sequences (28,240) aligned with ≥ 80% identity. Due to the same name prefix of the 13 individuals (HG), these unmapped sequences may be the bench-side contamination rather than real human sequences. Thus, we removed these sequences, re-computed the 90 people's novel sequence sizes (Supplementary Fig. 5) and re-aligned the novel sequences of 90 individuals to our common sequences.

Note 10: Commands and parameters

- Align reads to reference
```
bwa index -p ref GRCh38_primary.fa
bwa mem ref read1.fq read2.fq > alignment.sam
```

- Extract unaligned reads and corresponding mates
```
samtools fastq -f 12 alignment.sam -1 R1_Unalignedmate.fq -2 R2_Unalignedmate.fq
samtools fastq -f 68 -F 8 alignment.sam > R1_alignedmate.fq
samtools fastq -f 132 -F 8 alignment.sam > R2_alignedmate.fq
samtools view -f 8 -F 4 alignment.sam > alignedmate_GRCh38.sam
```

- Assemble unaligned reads into contigs
```
megahit -r R1_Unalignedmate.fq , R2_Unalignedmate.fq, R1_alignedmate.fq, R2_alignedmate.fq  -o sample_1
```

- Remove contaminations and contigs aligned to reference
```
makeblastdb -in contaminations.fa -dbtype nucl -out contamination
blastn -db contamination -query contig.fa -outfmt 6 -max_target_seqs 1  -max_hsps 1  -out  contig_contamination.tsv

makeblastdb -in GRCh38_alt.fa -dbtype nucl -out ref_alt_Id
blastn -db ref_alt_Id -query contig.fa -outfmt 6 -max_target_seqs 1  -max_hsps 1  -out  contig_ref.tsv
```

- Align reads to contigs
```
bowtie2-build filteredcontig.fa contig_Id
```

```
bowtie2 -x contig_Id -U R1_alignedmate.fq, R2_alignedmate.fq -S readtocontig.sam
```

- Determine the placement region by reads and mates

```
samtools view -h -F 2304 readtocontig.sam | samtools sort -n -O bam | bedtools bamtobed -i stdin | awk '{OFS="\t"} {print $4,$1,$6,$2,$3}' | sed -e 's/\/[1-2]//g' |sort > readtocontig.txt

samtools view -H alignedmate_GRCh38.sam | cat - <(awk 'NR==FNR{ a[$1]; next }$1 in a{ print $0 ; delete a[$1]; next }' readtocontig.txt <( samtools view alignedmate_GRCh38.sam )) | samtools sort -n -O bam | bedtools bamtobed -i stdin | awk '{OFS="\t"}{print $4,$1,$6,$2,$3}' | sed -e 's/\/[1-2]//g' | sort > pass_mates.txt

join -j 1 readtocontig.txt pass_mates.txt > mates_region.txt

samtools faidx GRCh38_no_alt.fa" region > GRCh38_Region.fa
nucmer --maxmatch -l 15 -b 1 -c 15 -p alignment_contig GRCh38Regions.fa end_contig.fa
```

- Cluster placed contigs

```
bedtools merge -d 20 -c 4 -o distinct -i placed_contigs.sorted.bed > merge_contigs.bed
```

- Remove contigs with no alignments to representatives

```
nucmer -p align_info  rep.fa cluster.fa
```

- Align other types of contigs to sequences in current clusters

```
makeblastdb -in remaining_cluster.fa -dbtype nucl -out remainingcontigs_Id
blastn -db remainingcontigs_Id -query othertype_contig.fa -outfmt 6 -max_target_seqs 1 -max_hsps 1 -out othertype_contig.tsv
```

- Merge left-end placed and right-end placed contigs into a longer insertion

```
nucmer -f  -p align_info left_placed.fa  right_placed.fa
delta-filter -q  -r -g -m -1 align_info > filterdalign_info.delta
show-coords -H -T -l -c -o filterdalign_info.delta > filterdalign_info.coords
popins merge -c left_right.fa
```

- Remove the redundancy of placed contigs

```
makeblastdb -in all_placed.fa -dbtype nucl -out all_placed_Id
blastn -db all_placed_Id -query all_placed.fa -outfmt 6 -max_target_seqs 1 -max_hsps 1 -out all_placed_aligned.tsv
```

- Cluster the unplaced contigs

```
cd-hit-est -i remain_unplaced.fa -o unplaced_cluster  -c 0.9 -n 8
```

*Additional programs used to analyze CPG

- Annotate placed contigs

```
vep -i contig_insertion_points.vcf -o contig_annotation --dir Cache_path --cache
--offline --fasta GRCh38_primary.fa --species homo_sapiens --everything --plugin
StructuralVariantOverlap,file=gnomad_v2_sv.sites.vcf.gz
```

- Compare with other genomes

```
bwa index -p other_genome_Id  other_genome.fa
bwa mem other_genome_Id CPG.fa > alignment.sam
```

- Call variants

```
bwa index -p new_ref_Id  new_ref.fa
bwa mem new_ref_Id read1.fq read2.fq > alignment.sam
java  -jar  picard.jar  MarkDuplicates  I=alignment.sam  O=alignment.markdup.sam
M=alignment.markdup.txt
java  -jar picard.jar BuildBamIndex I=alignment.markdup.sam
gatk HaplotypeCallerSpark -R GRCh38_decoy.fa -I alignment.markdup.sam -O vcffile
```

Supplementary Methods

Merging of single-end-placed contigs.
If the distance between the placement points of a LEP and a REP representative was at most 100 bp, we aligned the two representative contigs by NUCmer and ran show-coords -o to get the alignment result. The result was classified into four types.

1) When representatives of two clusters were identical or one contained another with ≥ 97% identity, the clusters were combined together and the longest contig became a new representative. The merged clusters became a BEP cluster.

2) If the alignment identity was between 90% and 97% and the insertion point of the LEP representative was to the left of the REP one on the same strand, the two clusters were combined using *PopIns merge* to get a new representative. The merged clusters became a BEP cluster.

3) If the coverage of either the LEP or REP representative was above 50% and at least one contig was shared by the two clusters, we merged them and chose the longest contig as a new representative.

4) If alignment between two representatives (an LEP and an REP) reached at least 90% identity and the insertion point of the REP representative was to the left of the LEP one, the two representatives were merged by *PopIns* and their clusters became a new SEP cluster.

Admixture analysis.
We called variants in the raw reads of the 486 individuals by GATK HaplotypeCaller (version 4.1.3.0) [12] and extracted the variants on chromosome 1. After carrying out linkage-disequilibrium-based pruning by PLINK version 20191028 (–indep-pairwise 1000 100 0.2) [13], we performed an unsupervised ADMIXTURE analysis to obtain an overview of the population compositions of the 486 individuals (Fig. 1A).

Supplementary References

1. Kim D, Song L, Breitwieser FP, Salzberg SL. Centrifuge: rapid and sensitive classification of metagenomic sequences. *Genome research* **26**, 1721-1729 (2016).

2. Breitwieser FP, Pertea M, Zimin AV, Salzberg SL. Human contamination in bacterial genomes has created thousands of spurious proteins. *Genome research* **29**, 954-960 (2019).

3. Sherry ST*, et al.* dbSNP: the NCBI database of genetic variation. *Nucleic acids research* **29**, 308-311 (2001).

4. El-Gebali S*, et al.* The Pfam protein families database in 2019. *Nucleic acids research* **47**, D427-D432 (2018).

5. Cantarel BL*, et al.* MAKER: an easy-to-use annotation pipeline designed for emerging model organism genomes. *Genome research* **18**, 188-196 (2008).

6. Montgomery SB*, et al.* Transcriptome genetics using second generation sequencing in a Caucasian population. *Nature* **464**, 773 (2010).

7. Kim D, Langmead B, Salzberg SL. HISAT: a fast spliced aligner with low memory requirements. *Nature methods* **12**, 357 (2015).

8. Zook JM*, et al.* Extensive sequencing of seven human genomes to characterize benchmark reference materials. *Scientific data* **3**, 160025 (2016).

9. Picard toolkit. *Broad Institute, GitHub repository*, (2019).

10. Wood D. Kraken 2 GitHub repository.).

11. Kozarewa I, Ning Z, Quail MA, Sanders MJ, Berriman M, Turner DJ. Amplification-free Illumina sequencing-library preparation facilitates improved mapping and assembly of (G+C)-biased genomes. *Nature methods* **6**, 291 (2009).

12. McKenna A*, et al.* The Genome Analysis Toolkit: a MapReduce framework for analyzing next-generation DNA sequencing data. *Genome research* **20**, 1297-1303 (2010).

13. Purcell S*, et al.* PLINK: a tool set for whole-genome association and population-based linkage analyses. *The American journal of human genetics* **81**, 559-575 (2007).
