# Supplementary material for: Building a Chinese pan-genome of 486 individuals
Source: Commun Biol. 2021 Aug 30;4:1016. doi: 10.1038/s42003-021-02556-6 (PMC8405635; doi:10.1038/s42003-021-02556-6)
Supplement: Supplementary file 3 — Description of Supplementary Files [file 42003_2021_2556_MOESM3_ESM.pdf]

Description of Additional Supplementary Files

File Name: Supplementary Data 1-9
Description:

Supplementary Data 1: A list of the placed sequences, with sequence ID, length, alignment details, and number of individuals having the sequence.

Supplementary Data 2: A list of the unplaced sequences, with sequence ID, length, and the number of individuals having the sequence.

Supplementary Data 3: Alignment of the novel sequences against the NCBI Nucleotide database by BLASTN.

Supplementary Data 4: Classification of the contaminations found in the 486 individuals.

Supplementary Data 5: Pathogenicity score of the sequences with a placement computed based on the CADD C-score.

Supplementary Data 6: VEP annotation of the insertion points.

Supplementary Data 7: Protein family classification using the RefSeq human protein database.

Supplementary Data 8: Annotation of the novel sequences using the Pfam database.

Supplementary Data 9: Repetitive sequence classification of the placed and unplaced sequences.
