# Supplementary material for: Building a Chinese pan-genome of 486 individuals
Source: Commun Biol. 2021 Aug 30;4:1016. doi: 10.1038/s42003-021-02556-6 (PMC8405635; doi:10.1038/s42003-021-02556-6)
Supplement: Supplementary file 5 — Reporting Summary [file 42003_2021_2556_MOESM5_ESM.pdf]

Corresponding author(s): Ruibang Luo, Ruiqiang Li, Tak-Wah Lam

Last updated by author(s): Jul 8, 2021

Reporting Summary

Nature Research wishes to improve the reproducibility of the work that we publish. This form provides structure for consistency and transparency in reporting. For further information on Nature Research policies, see our Editorial Policies and the Editorial Policy Checklist.

Statistics

For all statistical analyses, confirm that the following items are present in the figure legend, table legend, main text, or Methods section.

n/a	Confirmed	
☐	☒	The exact sample size (*n*) for each experimental group/condition, given as a discrete number and unit of measurement
☒	☐	A statement on whether measurements were taken from distinct samples or whether the same sample was measured repeatedly
☒	☐	The statistical test(s) used AND whether they are one- or two-sided<br>*Only common tests should be described solely by name; describe more complex techniques in the Methods section.*
☒	☐	A description of all covariates tested
☐	☒	A description of any assumptions or corrections, such as tests of normality and adjustment for multiple comparisons
☐	☒	A full description of the statistical parameters including central tendency (e.g. means) or other basic estimates (e.g. regression coefficient) AND variation (e.g. standard deviation) or associated estimates of uncertainty (e.g. confidence intervals)
☒	☐	For null hypothesis testing, the test statistic (e.g. *F*, *t*, *r*) with confidence intervals, effect sizes, degrees of freedom and *P* value noted<br>*Give P values as exact values whenever suitable.*
☒	☐	For Bayesian analysis, information on the choice of priors and Markov chain Monte Carlo settings
☒	☐	For hierarchical and complex designs, identification of the appropriate level for tests and full reporting of outcomes
☒	☐	Estimates of effect sizes (e.g. Cohen's *d*, Pearson's *r*), indicating how they were calculated

Our web collection on statistics for biologists contains articles on many of the points above.

Software and code

Policy information about availability of computer code

Data collection	Commands and parameters are included in Supplementary Note 10.
Data analysis	Custom scripts used are available at https://github.com/HKU-BAL/CPG.

For manuscripts utilizing custom algorithms or software that are central to the research but not yet described in published literature, software must be made available to editors and reviewers. We strongly encourage code deposition in a community repository (e.g. GitHub). See the Nature Research guidelines for submitting code & software for further information.

Data

Policy information about availability of data

All manuscripts must include a data availability statement. This statement should provide the following information, where applicable:

- Accession codes, unique identifiers, or web links for publicly available datasets
- A list of figures that have associated raw data
- A description of any restrictions on data availability

The novel sequences of CPG have been deposited to the Genome Sequence Archive for Human (http://bigd.big.ac.cn/gsa-human/) at the BIG Data Center, Beijing Institute of Genomics, Chinese Academy of Sciences, under the accession number PRJCA003657, following the regulations of the Human Genetic Resources Administration of China (HGRAC). The novel sequences of 910 Africans in APG were downloaded from NCBI with accession number PDBU01000000. The assembly of HX1, KOREF1.0, and NA18507 were downloaded from NCBI with accession number GCA_001708065.2, GCA_001712695.1, and GCA_000005465.1, respectively. The raw reads of the 90 Han Chinese from BGI were downloaded from EBI with accession number PRJEB11005.

Field-specific reporting

Please select the one below that is the best fit for your research. If you are not sure, read the appropriate sections before making your selection.

☒ Life sciences ☐ Behavioural & social sciences ☐ Ecological, evolutionary & environmental sciences

For a reference copy of the document with all sections, see nature.com/documents/nr-reporting-summary-flat.pdf

Life sciences study design

All studies must disclose on these points even when the disclosure is negative.

Sample size	We selected whole genome sequencing datasets from a pool of short-read sequencing datasets of normal Han Chinese individuals from the Novo-Zhonghua Genome Database. It is a commercial reference database of Chinese by Novogene Co., Ltd., Beijing, China, and has been used for multiple studies. Other than ethnicity as "Han Chinese", all individuals were de-identified and personal details were unavailable. In total, 486 individuals were employed in this study, with an average sequencing depth of 53.6-fold (min 27.1-fold, max 121.3-fold).
Data exclusions	We used verifyBamID to detect sample contamination and removed any individuals with an "estimate of contamination" greater than 0.03. We then analyzed the remaining 1000 genome project individuals using Principal Component Analysis and excluded any that significantly deviated from the CHB (Han Chinese in Beijing) group.
Replication	The constructed CPG were compared to HX1 (Chinese), KOREF1.0 (Korean) and NA18507 (African) for verification.
Randomization	n/a
Blinding	n/a

Reporting for specific materials, systems and methods

We require information from authors about some types of materials, experimental systems and methods used in many studies. Here, indicate whether each material, system or method listed is relevant to your study. If you are not sure if a list item applies to your research, read the appropriate section before selecting a response.

Materials & experimental systems

n/a	Involved in the study	
☒	☐	Antibodies
☒	☐	Eukaryotic cell lines
☒	☐	Palaeontology and archaeology
☒	☐	Animals and other organisms
☒	☐	Human research participants
☒	☐	Clinical data
☒	☐	Dual use research of concern

Methods

n/a	Involved in the study	
☒	☐	ChIP-seq
☒	☐	Flow cytometry
☒	☐	MRI-based neuroimaging
